# Changes in the Metabolic Profile of Melatonin Synthesis-Related Indoles during Post-Embryonic Development of the Turkey Pineal Organ

**DOI:** 10.3390/ijms231810872

**Published:** 2022-09-17

**Authors:** Kamila Martyniuk, Maria Hanuszewska-Dominiak, Bogdan Lewczuk

**Affiliations:** Department of Histology and Embryology, Faculty of Veterinary Medicine, University of Warmia and Mazury in Olsztyn, Oczapowskiego 13, 10-719 Olsztyn, Poland

**Keywords:** pineal organ, melatonin, serotonin, indoles, development, turkey, birds

## Abstract

Research on age-dependent changes in pineal activity has been limited almost exclusively to melatonin (MLT). This study determined, for the first time, the alterations occurring in the metabolic profile of MLT synthesis-related indoles during the post-embryonic development period in birds. Turkeys reared under a 12 h light/dark cycle were euthanized at 2 h intervals for 24 h at the ages of 2, 7, 14, and 28 days and 10, 20, 30, and 45 weeks. The results showed prominent changes in the metabolic profile of indoles during development and could be distinguished in four stages. The first stage, from hatching to the age of 2 weeks, was characterized by a decrease in the 5-hydroxytryptophan concentration and an increase in the concentrations of serotonin (5-HT), MLT, 5-methoxyindoleacetic acid, and 5-methoxytryptamine (5-MTAM). During the second stage, around the age of 1 month, the concentrations of N-acetylserotonin (NAS) and MLT reached a maximum. The synthesis and degradation of 5-HT were also the highest. The third stage, around the age of 10 weeks, was characterized by decreased levels of 5-HT (approximately 50%) and 5-hydroxyindoleacetic acid and a high level of 5-MTAM. The last stage, covering the age of 20 to 45 weeks, was characterized by a large decrease in the synthesis, content, and degradation of 5-HT. Despite these changes, there were no prominent differences in the nocturnal levels of NAS and MLT between the third and fourth stages. The concentrations of all tryptophan derivatives showed daily fluctuations until the age of 45 weeks.

## 1. Introduction

The mammalian pineal gland matures after birth [1], and the melatonin (MLT) rhythm appears during the postnatal period in both humans [2] and rodents [3,4]. In humans, the nocturnal peak of blood MLT reaches its highest levels between the ages of 3 and 5 years, then decreases until puberty, and remains at a relatively stable level up to the age of 35–40 years [5,6]. Thereafter, it progressively decreases, being the lowest after the age of 60. Rodents have a similar pattern of MLT secretion [7,8,9,10]. In rats, the serum MLT concentration shows an early developmental rise after birth followed by a high-level period from 10 to 30 days and further age-related reduction [7,8]. In contrast to its levels in the blood, the MLT content in the pineal gland of rats increases until adulthood [8]. As in humans, the decreases in the pineal and blood levels of MLT with aging are pronounced in rodents [7,8,9,10].

Few studies have investigated the age-dependent changes in MLT secretion in birds. In domestic chickens, the daily rhythms of MLT synthesis have been reported during embryonic life [11]. Plasma MLT concentrations rose in chickens from embryonic day 19 to post-embryonic day 3 and oscillated around this level to post-embryonic day 21 [12] or increased to post-embryonic day 14 [13]. An age-dependent increase in the level of circulatory MLT was observed in broilers from hatching to 42 days of age; however, the rate of this increase varied with age [14]. Similarly, the rhythmic synthesis of MLT begins during the embryonic life of the domestic goose [15]. A significant decline was reported in plasma MLT levels in old ring doves compared to the concentrations observed in mature and young animals [16]. Until now, studies on the levels of MLT in turkey blood have been focused on short life periods without considering possible changes in its levels at various stages of post-hatching development [17,18,19].

Research on age-dependent alterations in pineal activity has been limited almost exclusively to MLT. In the present study, we determined, for the first time, the changes occurring in the metabolic profile of MLT synthesis-related indoles during post-embryonic development. To achieve this goal, the activities of four enzymes involved in MLT synthesis and the content of ten indoles were measured in the same pineal organ.

This study was performed on domestic turkeys for three reasons. First, the domestic turkey is a perfect avian model for chronobiological research, with a high amplitude rhythm of MLT secretion and rapid, precise adaptation of this rhythm to changes in the light/dark cycle [20]. Second, the histology and ultrastructure of the pineal organ of turkeys are well characterized from day 1 to 1 year post-hatching life [21,22,23]. Morphological studies have shown that the pineal parenchyma is formed almost exclusively by rudimentary receptor pinealocytes up to the age of 4 weeks, and the involvement of secretory pinealocytes largely increases from the age of 20 weeks, changing the proportion between both types of cell [21,22]. This phenomenon enables the analysis of indole metabolism depending on the cellular composition of the parenchyma. Third, knowledge of domestic turkey physiology is important from a utilitarian point of view because of the great significance of turkeys as meat-producing animals [24,25]. Light is a critical factor that influences the health and production efficiency of poultry farms [26,27,28,29,30,31,32,33,34,35]. Many experiments have demonstrated the significance of MLT in mediating the effects of light on turkey productivity [26,27].

To characterize the changes in pineal indole metabolism during post-embryonic development, turkeys were reared from day 1 to 45 weeks under a 12 h light/dark cycle. The birds were euthanized at 2 h intervals for 24 h at the ages of 2, 7, 14, and 28 days and 10, 20, 30, and 45 weeks.

## 2. Results

### 2.1. Activities of Enzymes of the MLT Synthesis Pathway

Enzyme activity was expressed per 0.1 mg of the soluble protein content in the pineal homogenate. Two-way analysis of variance showed a significant effect of animal age on the activity of all MLT-synthesizing enzymes. Moreover, the effect of sampling time was significant in the analyses of data from tryptophan hydroxylase (TPH) and arylalkylamine N-acetyltransferase (AA-NAT) measurements. In the cases of both enzymes, significant interactions between animal age and sampling time were found.

#### 2.1.1. Tryptophan Hydroxylase

**Figure 1:**
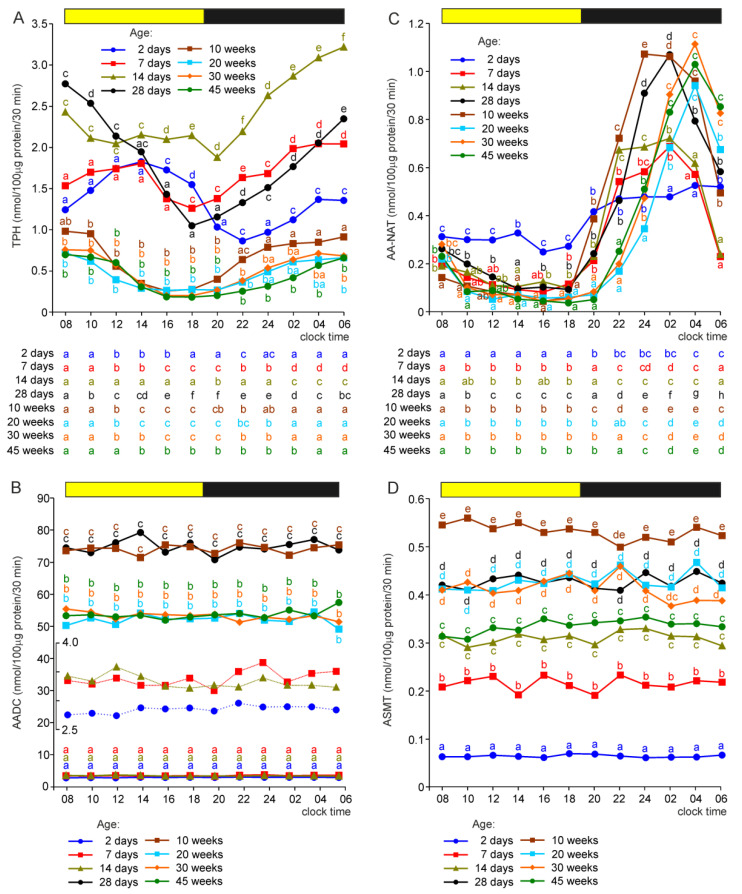
Activity (means) of tryptophan hydroxylase, TPH (**A**), aromatic amino acid decarboxylase, AADC (**B**), arylalkylamine N-acetyltransferase, AA-NAT (**C**), and N-acetylserotonin O-methyltransferase, ASMT (**D**) in the pineal organs of turkeys at the ages of 2, 7, 14, 28 days and 10, 20, 30, 45 weeks. The horizontal bar represents the periods of light and dark phases of the daily cycle. The letters on the charts show differences between age groups at each time-point in Duncan’s test. The letters below the charts show differences between time-points within a group in Duncan’s test. The same letters indicate means which are not significantly different. Insert in figure (**B**) shows AADC activity in the pineal organs of turkeys aged 2, 7, and 14 days at the limited *y*-axis scale.

The pineal activity of TPH (expressed per unit of the soluble protein content) increased within the first 2 weeks of post-hatching life (Figure 1A). During the scotophase, the TPH activity was significantly higher in 7-day-old turkeys than in 2-day-old turkeys and in 14-day-old birds than in 7-day-old birds. During the photophase, it was significantly higher in 14-day-old turkeys than in the younger birds. The activity of TPH in the pineal organs of turkeys aged 28 days was within the range defined by the levels found in the glands of birds aged 7 and 14 days. Compared to turkeys aged 28 days, TPH activity in birds aged 10, 20, 30, and 45 weeks was always significantly lower. Moreover, during the scotophase (from 22:00 h) and early photophase, it was significantly lower in the pineal organs of 45-week-old turkeys than in those of 10-week-old birds.

The TPH activity in the pineal organs of all studied groups of turkeys changed daily, but the course of these changes differed depending on animal age (Figure 1A). In the pineal organs of birds aged 2 and 7 days, the enzyme activity increased gradually during the first half of the photophase, slowly decreased during the second half, and then increased during the scotophase. In the pineal organs of birds aged 14 days, the TPH activity was rather stable during the photophase and increased prominently during the scotophase. The activity of TPH in older animals decreased gradually during the photophase and increased during the scotophase. The highest TPH activities were noted in these birds at 08:00, 10:00, 04:00, and 06:00 h and the lowest at 16:00 and 18:00 h.

#### 2.1.2. Aromatic Acid Decarboxylase (AADC)

The activity of AADC showed exceptional changes during the post-hatching development of the turkey pineal organ (Figure 1B). The lowest activities of this enzyme were found in turkeys at the age of 2 days. The AADC activities were approximately 25% higher in the pineal organs of birds aged 7 and 14 days. A 20-fold increase in the AADC activity was observed between the ages of 14 and 28 days. The activity of AADC in 10-week-old turkeys was similar to that in 4-week-old birds. A significant, approximately 30%, decrease in the AADC activity was noted between the ages of 10 and 20 weeks. No significant differences were found in the enzyme activity between the animals aged 20, 30, and 45 weeks. AADC showed no significant daily variations in its activity.

#### 2.1.3. Arylalkylamine N-Acetyltransferase

The effect of age on AA-NAT activity should be analyzed together with time-dependent changes in enzyme activity to avoid false conclusions (Figure 1C). In the pineal organs of turkeys of all age groups (except the youngest one), the activity of AA-NAT decreased at the beginning of the photophase and remained stable until the scotophase. AA-NAT activity increased after the onset of darkness, and this increase was slower in turkeys aged 20, 30, and 45 weeks than in younger birds. Consequently, the AA-NAT activity was lower in older animals than in younger ones at the beginning of the scotophase, and the nocturnal peak of AA-NAT activity occurred later in turkeys aged 20, 30, and 45 weeks than in younger birds. However, there were no differences in the peak activity of AA-NAT between the turkeys at 4–45 weeks. The nocturnal increase in AA-NAT activity was significantly lower in turkeys aged 2, 7, and 14 days than in older turkeys. It was also lower in 2-day-old birds than in 7- and 14-day-old turkeys. During the photophase, the AA-NAT activity was significantly higher in 2-day-old turkeys than in older birds.

#### 2.1.4. Serotonin O-Methyltransferase (ASMT)

The pineal activity of ASMT was the lowest in turkeys at the age of 2 days. It was approximately four-fold higher in 7-day-old birds and five-fold higher in 14-day-old birds than in the youngest individuals (Figure 1D). The ASMT activity was also significantly higher in turkeys aged 28 days than in birds at the age of 14 days. A further increase in AMST activity occurred between the ages of 4 and 10 weeks, resulting in the highest activity level at the end of this period. ASMT activity was significantly lower in the pineal organs of turkeys at 20, 30, and 45 weeks than in birds aged 10 weeks. No significant daily fluctuations in ASMT activity were found in any of the investigated groups of turkeys.

### 2.2. Indoles Related to Melatonin Synthesis Pathway

The concentration of indoles was expressed per 0.1 mg of the soluble protein content of the pineal homogenate. Two-way analysis of variance showed significant effects of animal age and sampling time on the pineal concentrations of all tryptophan derivatives. Significant interactions between these two factors were also observed for these compounds.

#### 2.2.1. Tryptophan (TRP)

**Figure 2:**
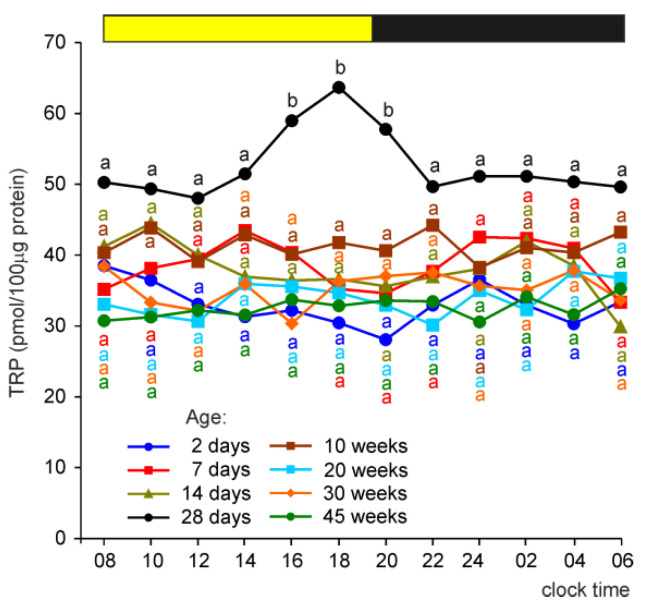
Concentration (means) of tryptophan in the pineal organs of turkeys at the ages of 2, 7, 14, 28 days and 10, 20, 30, 45 weeks. The horizontal bar represents the periods of light and dark phases of the daily cycle. The letters on the charts show differences between age groups at each time-point in Duncan’s test. The same letters indicate means which are not significantly different.

The concentration of TRP (expressed per unit of the soluble protein content) in the pineal organ was significantly higher between 16:00 and 20:00 h in turkeys at the age of 28 days than in younger and older birds (Figure 2). No significant daily fluctuations were observed in TRP levels independent of animal age.

#### 2.2.2. 5-Hydroxytryptophan (5-HTRP)

**Figure 3:**
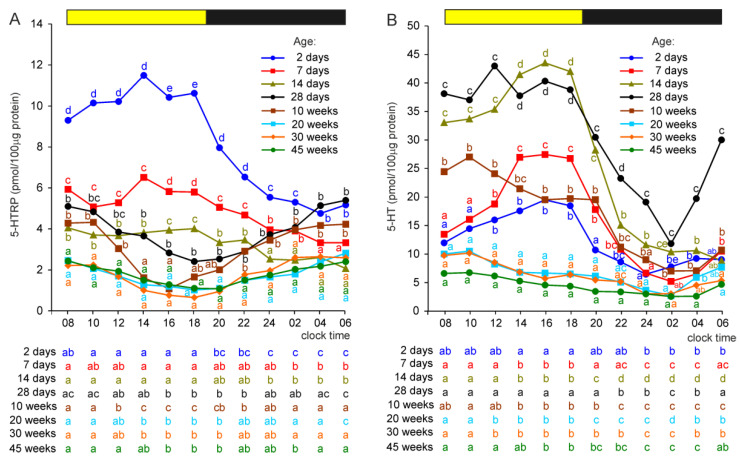
Concentrations (means) of 5-hydroxytryptophan, 5-HTRP (**A**), and serotonin, 5-HT (**B**), in the pineal organs of turkeys at the ages of 2, 7, 14, 28 days and 10, 20, 30, 45 weeks. The horizontal bar represents the periods of light and dark phases of the daily cycle. The letters on the charts show differences between age groups at each time-point in Duncan’s test. The letters below the charts show differences between time-points within a group in Duncan’s test. The same letters indicate means which are not significantly different.

The highest concentrations of 5-HTRP were found in the pineal organs of 2-day-old turkeys (Figure 3A). In the pineal organs of birds aged 7 days, 5-HTRP levels were significantly lower compared to those found in the youngest turkeys at all investigated time-points. Similarly, 5-HTRP concentrations were significantly lower in turkeys at the age of 14 days than in the birds aged 7 days (except at 04:00 and 06:00 h). More complex relationships were found when comparing the pineal concentrations of 5-HTRP in turkeys at the ages of 4 and 10 weeks with younger birds, mainly because of different courses of daily fluctuations. Generally, these concentrations were significantly lower at 16:00 and 18:00 h in 4- and 10-week-old turkeys than in younger ones but were similar or even higher at other time-points. The lowest concentrations of 5-HTRP were found in the pineal organs of turkeys at the ages of 20, 30, and 45 weeks. During the photophase, 5-HTRP concentrations were significantly lower in these turkeys than in birds at the ages of 2, 7, 14, and 28 days. During the scotophase (from 22:00 h), they were lower than those in turkeys at the ages of 2 and 28 days and 10 weeks.

Significant daily fluctuations in the concentration of 5-HTRP were found in all investigated groups of turkeys, but their courses and amplitudes differed markedly depending on the age of the animals (Figure 3A). In the pineal organs of 2-, 7-, and 14-day-old turkeys, 5-HTRP concentrations were significantly higher during the photophase than at 02:00, 04:00, and 06:00 h; however, the amplitudes of daily fluctuations decreased with the age of turkeys. In contrast, in older birds, the lowest levels of 5-HTRP were observed in the second half of the photophase. Among these animals, the highest amplitude of daily change was noted in 10-week-old turkeys.

#### 2.2.3. Serotonin (5-HT)

The concentration of 5-HT measured during the photophase consequently increased in the course of post-hatching development from the age of 2 to 14 days (Figure 3B). Such changes were not noted for the levels measured during the scotophase. 5-HT concentration in the pineal organs of the 28-day-old turkey was similar to that in 14-day-old birds. The level of 5-HT was significantly lower in the pineal organs of turkeys aged 10 weeks than in those of 28-day-old turkeys. The lowest concentrations of 5-HT were observed in turkeys at the ages of 20, 30, and 45 weeks. From 08:00 to 20:00 h, they were more than two-fold lower than those at the age of 10 weeks.

The concentration of 5-HT showed significant daily changes in all investigated groups. In the pineal organs of turkeys at the ages of 2, 7, and 14 days, the concentration of 5-HT increased during the photophase to reach maximal values in the second half of this phase and then decreased during the scotophase (Figure 3B). The amplitude of daily fluctuations increased with the age of the birds. In 28-day-old turkeys, the level of 5-HT was rather stable during the photophase and decreased after the onset of the scotophase, reaching the minimum value at 02:00, and then quickly increased. The concentration of 5-HT in older birds was the highest at 08:00 and 10:00 h, followed by a gradual decrease to reach the minimum at 02:00 h, and then increased again.

#### 2.2.4. N-Acetylserotonin (NAS)

**Figure 4:**
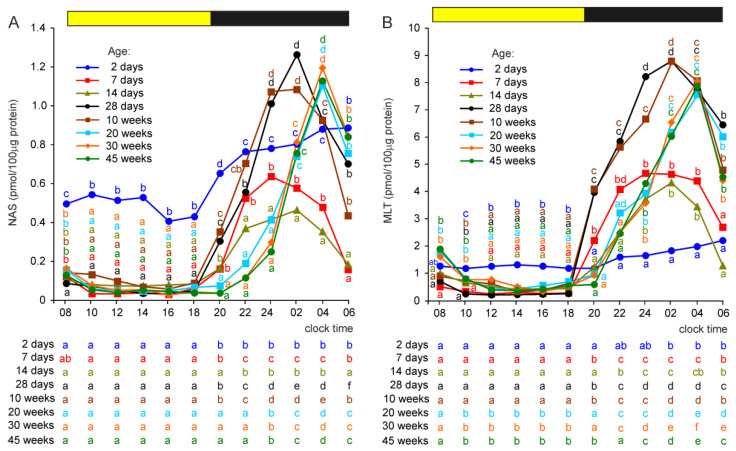
Concentrations (means) of N-acetylserotonin, NAS (**A**), and melatonin, MLT (**B**), in the pineal organs of turkeys at the ages of 2, 7, 14, 28 days and 10, 20, 30, 45 weeks. The horizontal bar represents the periods of light and dark phases of the daily cycle. The letters on the charts show differences between age groups at each time-point in Duncan’s test. The letters below the charts show differences between time-points within a group in Duncan’s test. The same letters indicate means which are not significantly different.

The photophase concentrations of NAS were approximately 3–5-fold higher in 2-day-old turkeys than in older birds (Figure 4A). During the scotophase, NAS concentrations increased significantly in all groups of turkeys. In the initial phase of post-hatching development, the scotophase levels of NAS decreased with age; they were significantly lower in 7-day-old birds than in 2-day-old and in 14-day-old than in 7-day-old birds. In the next period of post-hatching development, the scotophase concentrations of NAS increased and were significantly higher in turkeys aged 4, 10, 20, 30 and 45 weeks than in 2-, 7-, and 14-day-old birds.

The daily profiles of NAS concentrations differed markedly between age groups. In turkey aged 2 days, the NAS level increased approximately two-fold within 4 h after the onset of darkness and then remained at a relatively stable level (Figure 4A). In older birds, the levels of NAS increased stepwise in the first part of the scotophase and then decreased. The peaks were reached earlier in turkeys aged 7, 14, and 28 days and 10 weeks than in older birds. 

#### 2.2.5. Melatonin

The concentration of MLT was significantly higher during the photophase between 10:00 and 18:00 h in turkeys aged 2 days than in older birds, but the differences were much smaller compared to those in the NAS level (Figure 4B). During the scotophase, between 22:00 and 04:00 h, MLT concentration was significantly lower in 2-day-old turkeys than in older birds. The levels of MLT in the pineal organs of birds aged 7 and 14 days were mostly similar and approximately two-fold higher than in 2-day-old turkeys and approximately two-fold lower compared to the peak values in birds aged 4–45 weeks. The daily profiles of MLT concentration were similar to those of the NAS levels. 

#### 2.2.6. 5-Hydroxyindole Acetic Acid (5-HIAA) and 5-Hydroxytryptophol (5-HTOL)

**Figure 5:**
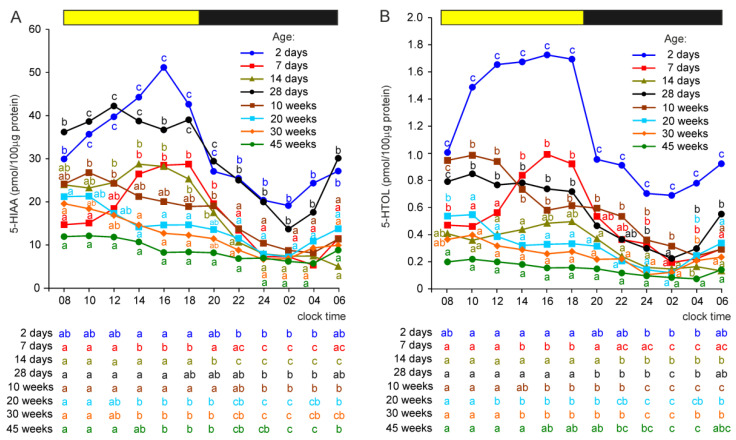
Concentrations (means) of 5-hydroxyindole acetic acid, 5-HIAA (**A**), and 5-hydroxytryptophol, 5-HTOL (**B**), in the pineal organs of turkeys at the ages of 2, 7, 14, 28 days and 10, 20, 30, 45 weeks. The horizontal bar represents the periods of light and dark phases of the daily cycle. The letters on the charts show differences between age groups at each time-point in Duncan’s test. The letters below the charts show differences between time-points within a group in Duncan’s test. The same letters indicate means which are not significantly different.

In contrast to 5-HT levels, the concentration of 5-HIAA was significantly higher in the pineal organs of turkeys at the age of 2 days than in 7- and 14-day-old birds (Figure 5A). The level of 5-HIAA in 28-day-old birds was similar to that in 2-day-old turkeys. In animals aged 10, 20, 30, and 45 weeks, the pineal concentrations of 5-HIAA were significantly lower than in turkeys aged 28 days.

The concentrations of 5-HIAA showed significant daily changes in all turkey groups, and their courses were similar to the age-specific daily fluctuations in the 5-HT level.

The pineal level of 5-HTOL decreased during development between 2 and 14 days of age, with a prominent drop between 2 and 7 days (Figure 5B). Next, it increased in turkeys at the ages of 4 and 10 weeks compared to the concentrations found in 14-day-old birds, but significant differences were not noted at all time-points because of the different course of the daily profile of 5-HTOL in these groups. The concentrations of 5-HTOL were significantly lower between 08:00 and 02:00 h in turkeys at the ages of 20, 30, and 45 weeks than in birds at the age of 10 weeks. 

The concentrations of 5-HTOL significantly changed during the daily cycle. The course of these changes differed depending on the age of the birds and was similar to that of 5-HT levels.

#### 2.2.7. 5-Methoxyindole Acetic Acid (5-MIAA) and 5-Methoxytryptophol (5-MTOL)

**Figure 6:**
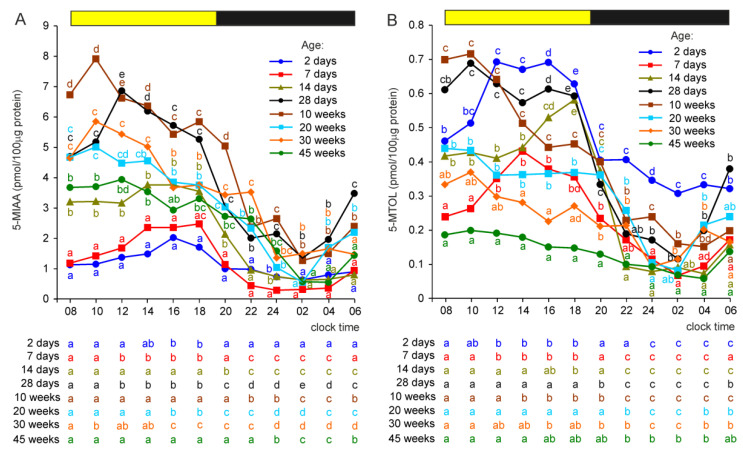
Concentrations (means) of 5-methoxyindole acetic acid, 5-MIAA (**A**), and 5-methoxytryptophol, 5-MTOL (**B**), in the pineal organs of turkeys at the ages of 2, 7, 14, 28 days and 10, 20, 30, 45 weeks. The horizontal bar represents the periods of light and dark phases of the daily cycle. The letters on the charts show differences between age groups at each time-point in Duncan’s test. The letters below the charts show differences between time-points within a group in Duncan’s test. The same letters indicate means which are not significantly different.

In contrast to 5-HIAA, the lowest concentrations of its metabolite, 5-MIAA, were found in 2- and 7-day-old turkeys (Figure 6A). The concentration of 5-MIAA was higher in birds at the age of 14 days than in younger birds between 08:00 and 20:00 h. In turkeys aged 4 and 10 weeks, the pineal levels of 5-MIAA were significantly higher than in younger birds at all time-points. The levels of 5-MIAA were significantly lower in 20-, 30-, and 45-week-old turkeys compared to 10-week-old birds during the photophase but not during the scotophase.

The concentrations of 5-MIAA changed significantly during the daily cycle in all groups of turkeys (Figure 6A). The concentrations of 5-MIAA in the pineal organs of 2-day-old turkeys were significantly higher at 16:00 and 18:00 h than between 20:00 and 12:00 h. In birds aged 7 and 14 days, they were significantly higher between 08:00 and 20:00 h than between 22:00 and 04:00 h (or 06:00 h in 14-week-old birds). The highest levels of 5-MIAA in these birds were noted in the second half of the photophase. In contrast, in older birds, the concentrations of 5-MIAA were the highest in the first half of the photophase. In 28-day-old turkeys, 5-MIAA concentrations were significantly higher during the photophase than during the scotophase, with a peak at 12:00 h and a nadir at 02:00 h. Similar daily profiles of 5-MIAA levels, but with a peak at 10:00 h, were found in 10-, 20-, and 30-week-old turkeys. In turkeys aged 45 weeks, the levels of 5-MIAA were only slightly higher in the first half of the photophase than in the second one, and the lowest concentrations of 5-MIAA were observed at 02:00 and 04:00 h.

The concentration of 5-MTOL decreased during the initial days after hatching and was significantly lower in 7-day-old turkeys than in those at the age of 2 days at all time-points (Figure 6B). Subsequently, it increased and exhibited more prominent daily fluctuations. In turkeys at the ages of 14 and 28 days and 10 weeks, 5-MTOL concentrations reached the levels found in 2-day-old birds during the photophase; however, they were much lower than in 2-day-old turkeys during the scotophase. Compared with 10-week-old turkeys, the concentrations of 5-MTOL were significantly lower in birds at the ages of 20, 30, and 45 weeks during the entire photophase. Significant differences in the level of 5-MTOL were also noted between the birds aged 20 and 45 weeks during the photophase.

Daily fluctuations in 5-MTOL concentration, with higher levels during the photophase than in the scotophase, were noted in all investigated groups of turkeys (Figure 6B). The course of these fluctuations differed between the age groups. In 2-, 7-, and 14-day-old turkeys, 5-MTOL concentrations were higher in the second half than in the first half of the photophase, while in older animals the highest levels of 5-MTOL were noted shortly after the onset of the photophase. In all groups of turkeys, the level of this compound prominently declined after the onset of the scotophase.

#### 2.2.8. 5-Methoxytryptamine (5-MTAM)

**Figure 7:**
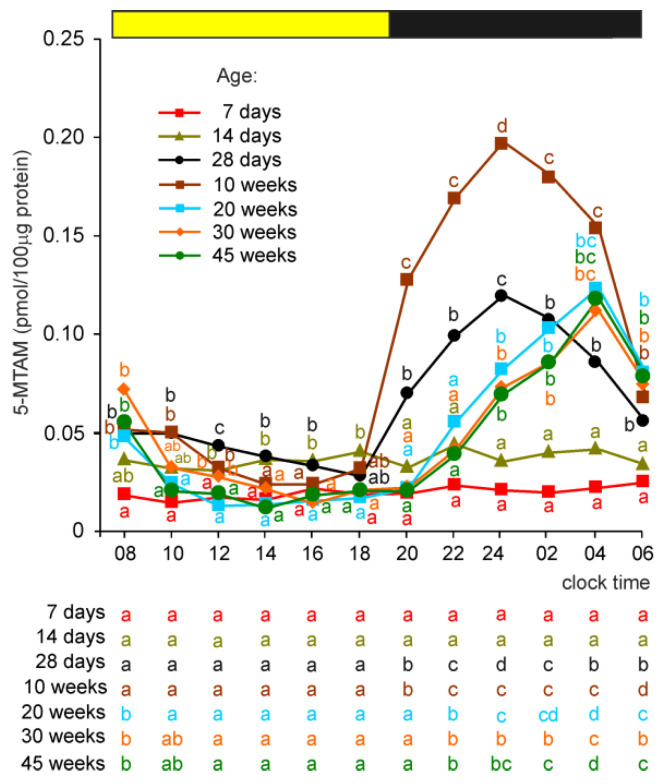
Concentration (means) of 5-methoxytryptamine, 5-MTAM, in the pineal organs of turkeys at the ages of 2, 7, 14, 28 days and 10, 20, 30, 45 weeks. The horizontal bar represents the periods of light and dark phases of the daily cycle. The letters on the charts show differences between age groups at each time-point in Duncan’s test. The letters below the charts show differences between time-points within a group in Duncan’s test. The same letters indicate means which are not significantly different. The concentration of 5-MTAM was undetectable in the pineal organs of turkeys aged 2 days.

5-MTAM was undetectable in the pineal organs of 2-day-old turkeys. The level of 5-MTAM was greater than the quantification limit in birds aged 7 and 14 days, being higher in older animals, but did not show daily fluctuations (Figure 7). The nighttime increase in 5-MTAM concentration was noted for the first time in 28-day-old turkeys. The scotophase level of this amine was higher in turkeys at the age of 10 weeks than in birds aged 28 days. In turkeys aged 20, 30, and 45 weeks, the nocturnal peak levels of 5-MTAM were similar to those in 28-week-old birds but occurred later than in younger birds.

## 3. Discussion

The present study reports for the first time that the metabolic profile of pineal indoles undergoes prominent changes during the post-embryonic development period of birds. Our experiment covered a period from hatching through sexual maturation (start of egg laying at the age of 24–25 weeks) to somatic maturity (age of 30 weeks) and also included 15 weeks of life thereafter. The phase of intensive growth (up to the age of 4 weeks) was analyzed in detail by collecting samples at the ages of 2, 7, 14, and 28 days.

Four stages could be distinguished in the course of post-embryonic changes in the metabolic profile of MLT synthesis-related indoles in the pineal organ of turkeys. The first stage covered the period from hatching to the age of 14 days and was characterized by large changes in the indole levels, including (i) a decrease in 5-HTRP concentration; (ii) an increase in 5-HT concentration; and (iii) an increase in the concentrations of MLT, 5-MIAA, and 5-MTAM. The decrease in the 5-HTPR level seems to be a result of its intensive utilization for 5-HT synthesis due to the increased activity of AADC. It occurs despite an approximately two-fold increase in nighttime activity of TPH between the ages of 2 and 14 days. The analysis of enzyme activity and indole content demonstrated that the increase in 5-HT concentration has two sources: (i) increased synthesis due to an elevation in the AADC activity and (ii) diminished degradation by oxidative deamination, as indicated by the decreased levels of 5-HIAA and 5-HTOL. The activity of monoamine oxidase (MAO) was not measured in our study; therefore, we cannot explain the mechanism responsible for the reduced degradation of 5-HT, and both the decreased activity of MAO and the development of 5-HT storage in a cell compartment unavailable for MAO should be considered. A study on the embryonic goose pineal organ showed that the unknown mechanism of 5-HT protection against degradation by MAO develops in embryonic life and is further improved in post-embryonic life [15]. The increase in 5-methoxyindole concentration is a result of a large, five-fold increase in the ASMT activity between the ages of 2 and 14 days. The apparent consequences of elevated ASMT activity are the decrease in NAS concentration and the increase in MLT concentration as well as the increase in 5-MIAA concentration. In contrast to NAS and MLT, the concentration of 5-HIAA was 10-fold higher than 5-MIAA; therefore, methylation intensity has no effect on 5-HIAA levels. Surprisingly, the level of 5-MTOL decreased between the ages of 2 and 7 days and then increased. The decrease could be explained by a large drop in 5-HTOL concentration in this period and the very high affinity of 5-HTOL to ASMT. The synthesis of 5-MTAM (at detectable levels) started during the first stage of development, but it did show daily fluctuations.

The second stage of post-embryonic development of pineal indole metabolism was defined by the results obtained in turkeys at the age of 28 days. At this stage, the activity of AA-NAT and the concentrations of NAS and MLT reached their maximum values. The activity of AADC was more than 20-fold higher in 28-day-old birds than in 14-day-old ones and also reached the highest level in our study. The concentrations of 5-HT in turkeys aged 14 and 28 days were similar and the highest among the investigated groups of turkeys. The concentrations of 5-HIAA, 5-HTOL, 5-MIAA, and 5-MTOL were much higher in 28-day-old birds than in 14-day-old turkeys, pointing to more intensive synthesis and degradation of 5-HT in this stage than in the previous one. The concentration of 5-MTAM was markedly higher than in younger birds and showed prominent daily changes, with a peak at 24:00 h. The level of TRP was also the highest in birds aged 28 days.

The metabolic profile of pineal indoles in turkeys aged 10 weeks was proposed by us as the third stage of post-embryonic changes. It was characterized by almost 50% lower concentrations of 5-HT and 5-HIAA compared to those in 28-day-old birds. In contrast, the concentrations of 5-MIAA were similar, which suggests an increase in the methylation capacity. The levels of NAS and MLT also did not vary between turkeys aged 28 days and 10 weeks. The concentration of 5-MTAM reached its highest level in our experiment. Regarding enzymes, the activity of TPH was lower than in younger turkeys, while there were no differences in AADC and AA-NAT activities between stages two and three. The ASMT activity was the highest measured in the present study.

The fourth stage covered the period from the age of 20 to 45 weeks, in which the metabolic profile showed rather small changes. The most characteristic feature of this period was prominently reduced synthesis, content, and degradation of 5-HT compared to those in the previous stages. The activity of TPH and the concentration of 5-HTRP were the lowest in these birds. The activity of AADC was reduced by approximately 25% compared to that in 4-week-old turkeys, while 5-HT concentration was approximately five- to ten-fold lower in these birds. The concentrations of 5-HIAA and 5-HTOL were approximately two- to four-fold lower than those in 4-week-old turkeys. The decreases in the 5-MIAA and 5-MTOL levels were lesser than those of their substrates, probably because the activity of ASMT was reduced only by 20%. The reduction in 5-HT metabolism progressed slowly from 20 to 45 weeks of age. It should be noted that, despite the changes in 5-HT concentrations, there were no prominent differences in the activity of AA-NAT and the levels of NAS and MLT at the nocturnal peaks. The maximum concentration of 5-MTAM was lower than that in 4- and 10-week-old birds.

The structural changes in the pineal organ of turkeys occurring during the period of post-hatching development have been investigated by light and electron microscopy [21,22]. The results of these studies have enabled us to correlate changes in the profile of pineal indoles with those in the pineal histology and ultrastructure. The turkey pineal organ is characterized by a follicular organization of parenchyma throughout the entire period of post-hatching life; however, the developmental changes affect many aspects of pineal morphology [21,22,36,37]. From the perspective of the present study, the most important are age-dependent transformations in the follicular wall architecture. The inner part of the follicle wall, surrounding the follicular lumen, is formed by rudimentary photoreceptor pinealocytes and ependymal-like supporting cells. The outer part of the follicle wall is composed of secretory pinealocytes, astrocyte-like cells, and cell processes and their endings. Up to the age of 4 weeks, the outer part of the follicle is limited to very few cells located close to the basement membrane; therefore, the follicles are formed almost exclusively by rudimentary photoreceptor pinealocytes and ependymal-like supporting cells. The number of cells in the outer part of the follicular wall is higher in 10-week-old turkeys but increased largely in 20-week-old birds. Next, the number of secretory pinealocytes and astrocyte-like cells continues to increase, causing a reversal in the proportion between the inner and outer parts of the follicle wall. Ultrastructural studies showed that rudimentary photoreceptor pinealocytes developed up to the age of 10 weeks and underwent regression after the age of 20 weeks [21,22]. The progressive processes in these cells were the most intensive during the first 4 weeks of post-embryonic life and included cell elongation, development of rough endoplasmic reticulum and Golgi apparatus, increase in the number of mitochondria, and formation of a regular stratified distribution of organelles. The regression of rudimentary photoreceptor pinealocytes was visible as a shortage of cells and a decrease in the number of organelles.

The first stage of changes in indole metabolism distinguished in the present study, during which cells increase their capability of 5-HT and MLT synthesis, correlates with an early phase of post-embryonic development of rudimentary receptor pinealocytes, characterized mainly by an increase in the number of rough endoplasmic reticulum cisterns and mitochondria. The second and third stages of post-embryonic changes in indole metabolism coincide with the presence of highly developed rudimentary receptor pinealocytes with a stratified distribution of organelles, numerous cisterns of rough endoplasmic reticulum, well-developed Golgi apparatus surrounded by abundant clear and dense core vesicles, and numerous mitochondria in their upper parts. Based on the present results, it can be concluded that well-developed rudimentary receptor pinealocytes are characterized by very high synthesis and metabolism of 5-HT. The rudimentary receptor pinealocytes are slightly better developed during the third stage than during the second stage. Moreover, secretory pinealocytes are more abundant during the third stage than during the second stage. Therefore, lower 5-HT concentrations during the third stage could be attributed to the final maturation of rudimentary receptor pinealocytes or the presence of more secretory pinealocytes. The fourth stage of changes in indole metabolism correlates with a large increase in the number of secretory pinealocytes and regression of rudimentary receptor pinealocytes. It is reasonable to assume that the synthesis and degradation of 5-HT are reduced in secretory pinealocytes, while MLT synthesis is kept at a similar level to that in rudimentary receptor pinealocytes. The concentrations of 5-HT and 5-HIAA decrease between 20 and 45 weeks of age, and, at the same time, there is an increase in the number of secretory pinealocytes and the regression of rudimentary receptor pinealocytes.

Analyzing the results of morphological studies, it should be noted that, although the quantitative ratios of pinealocytes to supporting cells have not been established, double immunofluorescence staining for glial fibrillary acidic protein (supporting cells marker) and ASMT clearly shows that the proportion between these cell populations does not change noticeably from the age of 14 days to 56 weeks [22]. Supporting cells were negative for the immunochemical reactions of TPH and 5-HT. Attention should be paid to the intensive development of connective tissue around the follicles and in the pineal capsule after the age of 20 weeks [21]. Considering that the proportion of connective tissue to parenchyma increases during development, we expressed our data per soluble protein content in homogenate, not per tissue weight, because the collagen fibers were not dissolved during sonication. In this way, it was possible to reduce the effect of increased connective tissue content on the results.

**Figure 8:**
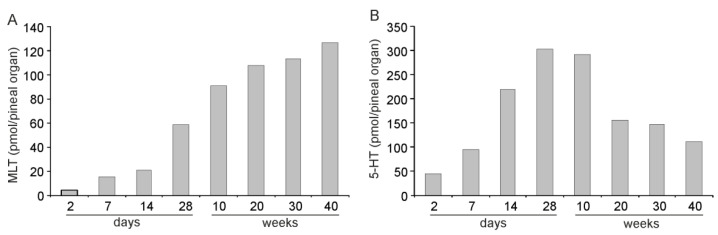
Contents (means) of melatonin, MLT (**A**), and serotonin, 5-HT (**B**), at their peak values in the pineal organs of turkeys at the ages of 2, 7, 14, 28 days and 10, 20, 30, 45 weeks.

From an endocrinological point of view, it would be interesting to know the changes in the total content of MLT in the pineal organ. The nocturnal peak value of MLT content increased with the age of animals up to the end of the experiment, most intensively between the ages of 2 and 10 weeks (Figure 8A). For comparison, the diurnal peak value of 5-HT content decreased markedly after the age of 10 weeks (Figure 8B).

The concentrations of all measured derivatives of TRP, except 5-MTMA, showed significantly daily fluctuations throughout the entire study period; however, the course of these fluctuations changed with the age of the turkeys. The data obtained from 2-day-old birds should be considered with a remark that newly hatched chicks were purchased from a commercial hatchery, where eggs were incubated without a strictly defined light/dark cycle. The use of laboratory-hatched chicks was not possible because of the large number of birds needed for the experiment. In all age groups, the daily fluctuations in indole concentrations were driven by alterations in TPH and AA-NAT activities. The course of daily changes in TPH activity changed prominently up to the age of 10 weeks and then stabilized. Generally, the morning decrease in TPH activity started later in young birds than in those aged 10 weeks and older. The nocturnal peaks of AA-NAT occurred with delay in turkeys aged 20, 30, and 45 weeks compared to that in the younger birds. There were also some differences in the shape of these peaks. The age-dependent changes in the daily profiles of 5-TPH, 5-HT, 5-HIAA, 5-HTOL, 5-MIAA, and 5-MTOL were highly variable because they were the results of alterations in the activity of both TPH and AA-NAT. In contrast, the changes in daily profiles of NAS and MLT followed the alteration in AA-NAT activity. The daily rhythm of 5-MTAM occurred from the age of 4 weeks. The mechanism of its generation is unknown. The nocturnal peak of 5-MTAM appeared 4 h later in turkeys aged 20, 30, and 45 days than in those aged 4 and 10 weeks.

The differences in the daily profiles of MLT synthesis enzymes and indoles between the turkeys at age 20–45 weeks and younger birds are most probably related to the increased number of secretory pinealocytes. It should be noted that pinopsin, a non-visual opsin, is present in the turkey pineal organ during the entire period of postnatal development, but its distribution shows prominent age-dependent changes, including a subsequent increase in pinopsin-positive processes in the outer part of the follicle wall and a reduction in the number and size of positive apical extensions [23]. These data suggest that secretory pinealocytes are photoreceptive. The studies performed in superfusion culture showed that the pineal organs of 45-week-old turkeys were less sensitive to light exposure at night than those of 10-week-old ones [23]. However, it should be underlined that the pineal organs of 45-week-old turkeys in vitro secrete melatonin in a well-entrained diurnal rhythm and adopt their secretory activity to a reversed light/dark cycle as fast as the pineal organs of 10-week-old turkeys and faster than the pineal organ of 15-week-old chickens [38], 12-week-old geese [39], and 9-month-old ducks [40]. The differences in daily profiles of indoles and enzymes involved in their synthesis between young and adult turkeys may also be related to the increased role of sympathetic regulation with the age of birds, as was suggested by the studies performed on domestic chickens [41]. Sympathetic innervation provides the signal from the suprachiasmatic nucleus that acts as a circadian oscillator controlled by the retina [42,43]. This signal can affect the rhythm generated by pinealocytes under the control of their light reception cascades.

## 4. Materials and Methods

### 4.1. Birds and Experimental Design

This study was performed on female domestic turkeys, Hybrid Converter strain (Hendrix Genetics, Boxmeer, The Netherlands), obtained from a commercial hatchery (Grelavi, SA, Olsztyn, Poland). One-day-old chicks were transported to the chronobiological animal laboratory at the Faculty of Veterinary Medicine, University of Warmia and Mazury in Olsztyn, Poland, where birds were reared for 45 weeks. The turkeys were kept under a 12 h light/dark cycle with a light intensity of 20 lx measured at the head level. The light was provided by tungsten lamps (3200 K), which were automatically switched on at 07:00 h and switched off at 19:00 h. The birds had free access to standard food and water. At the ages of 2, 7, 14, and 28 days, 5 turkeys were euthanized at 08:00, 12:00, 14:00, 16:00, 18:00, 20:00, 22:00, 24:00, 02:00, 04:00, and 06:00 h. At the ages of 10, 20, 30, and 45 weeks, 4 birds were sacrificed at the same time-points. The pineal organs were collected immediately and frozen at −75 °C. During the night, procedures were conducted in complete darkness using noctovision goggles. The experiment was performed as per Polish and EU law (Opinion of the Animal Welfare Board of the FVM, UWM in Olsztyn, 2 December 2019).

### 4.2. Assay of Enzyme Activity and Indole Content

#### 4.2.1. Sample Preparation

Frozen pineal glands were weighed and sonicated (5 × 2 s, 1 W) in 200 µL of cold water using a Vibra-Cell VC 70 ultrasonic processor equipped with a 2 mm probe (Sonics and Materials Inc., Newtown, CT, USA) in an ice bath. Then, 80 µL of homogenate was added to an Eppendorf tube containing 20 µL of 1 mM perchloric acid, and the mixture was vortexed and incubated for 15 min in an ice bath. After centrifugation at 30,000× *g* (4 °C) for 15 min (Allegra 64R, Coulter Beckman, Brea, CA, USA), the supernatant was carefully transferred into autosampler vials and used in the assay of MLT synthesis-related indoles. From the remaining part of the homogenate, 20 µL aliquots were added to Eppendorf tubes containing substrates used in the assays of TPH, AADC, AA-NAT, and ASMT. The time between the tissue sonication and the beginning of incubation in the enzyme assays did not exceed 30 s.

#### 4.2.2. Content of Melatonin Synthesis-Related Indoles

The indole content in the pineal organ homogenates was measured by HPLC with fluorescence detection [44] using a Vanquish Duo U/HPLC system equipped with two gradient pumps, a cooled dual split autosampler, column compartment, and two fluorescence detectors (Thermo Fisher Scientific, Waltham, MA, USA), as previously described [45].

#### 4.2.3. Measurement of the Melatonin Synthesis Pathway Enzymes’ Activities

##### TPH Activity

The activity of TPH was determined by measuring the accumulation of 5-HTP during incubation of the pineal homogenate with appropriate substrates and the AADC inhibitor [46] according to a previously described protocol [45].

##### AADC Activity

The activity of AADC was determined by measuring the decarboxylation of 5-HTP into 5-HT in the presence of a monoamine oxidase inhibitor [47] according to a previously described protocol [45].

##### AA-NAT Activity

The AA-NAT activity was determined by measuring the accumulation of N-acetyltryptamine [48] according to a previously described protocol [45].

##### ASMT Activity

ASMT activity was determined by measuring the accumulation of MLT [49] according to a previously described protocol [45].

### 4.3. Assay of Protein Content

The pineal homogenate was diluted in water and used to determine the protein content using the Quant-iT™ Protein Assay Kit.

### 4.4. Statistical Analysis

The data were analyzed using two-way analysis of variance (ANOVA) with age and sampling time as factors. Duncan’s test was used as a post hoc test. A *p*-value < 0.05 was considered statistically significant. The analyses were performed using Dell Statistica 13 (Version 13.1 PL, Dell Inc., Tulsa, OK, USA).

## 5. Conclusions

The concentration of MLT (expressed per unit of soluble protein content) at the nocturnal peak increased in the turkey pineal organ up to the age of 4 weeks and then remained at a rather constant level until the age of 45 weeks, when the study was finished. Because of pineal organ growth, the total content of MLT at the nocturnal peak increased with different intensities until the end of the experiment. However, the analysis of indoles other than MLT and enzymes involved in their synthesis revealed that, beyond this simple image of developmental changes, there are large transformations in the metabolism of pineal indoles.

Four stages could be distinguished in the course of the post-embryonic changes in the metabolic profile of MLT synthesis-related indoles. The first stage covers the period from hatching to the age of 2 weeks and is characterized by (i) the decrease in 5-HTRP concentration; (ii) the increase in 5-HT concentration; and (iii) the increase in the concentration of MLT, 5-MIAA, and 5-MTAM. During the second stage, around the age of 1 month, the activity of AA-NAT and the concentrations of NAS and MLT reach their maximal values. The synthesis and degradation of 5-HT at this stage are the highest among the investigated age groups of turkeys. The third stage, around the age of 10 weeks, is characterized by approximately 50% decreased concentrations of 5-HT and 5-HIAA. The levels of 5-MIAA are similar to those in the second stage, which is correlated with the highest activity of ASMT. The concentration of 5-MTAM also reaches the highest level. The last (fourth) stage covers a period from the age of 20 to 45 weeks and is characterized by extraordinarily decreased synthesis, content, and degradation of 5-HT. The changes in the metabolism of pineal indoles correlate with the development of rudimentary receptor pinealocytes (stages I and II) and secretory pinealocytes (stage IV). The obtained results have demonstrated that the concentrations of all measured derivatives of TRP show significant daily fluctuations up to the age of 45 weeks; however, the course of these fluctuations changed with the age of turkeys. The prominent shift in nocturnal peaks of AA-NAT, NAS, and MLT correlates with the predominance of secretory pinealocytes.

The obtained results show that the studies of indole metabolic profiles may provide many important data on developmental and aging processes occurring in the pineal gland, both in non-mammalian and mammalian species.

## Data Availability

The data presented in this study are available on request from the corresponding author.

## Abbreviations

5-HIAA	5-hydroxyindoleacetic acid
5-HT	serotonin
5-HTOL	5-hydroxytryptophol
5-HTRP	5-hydroxytryptophan
5-MIAA	5-methoxyindoleacetic acid
5-MTAM	5-methoxytryptamine
5-MTOL	5-methoxytryptophol
5-MTRP	5-methoxytryptophan
AADC	aromatic L-amino acid decarboxylase
AA-NAT	arylalkylamine N-acetyltransferase
ASMT	N-acetylserotonin O-methyltransferase
HPLC	high-pressure liquid chromatography
MLT	melatonin
NAS	N-acetylserotonin
TPH	tryptophan hydroxylase
TRP	tryptophan
